# The Involvement of Phospholipases A_**2**_ in Asthma and Chronic Obstructive Pulmonary Disease

**DOI:** 10.1155/2013/793505

**Published:** 2013-05-13

**Authors:** Ewa Pniewska, Rafal Pawliczak

**Affiliations:** Department of Immunopathology, Faculty of Biomedical Sciences and Postgraduate Training, Medical University of Lodz, 7/9 Zeligowskiego Street, Building 2, Room 122, 90-752 Lodz, Poland

## Abstract

The increased morbidity, mortality, and ineffective treatment associated with the pathogenesis of chronic inflammatory diseases such as asthma and chronic obstructive pulmonary disease (COPD) have generated much research interest. The key role is played by phospholipases from the A_2_ superfamily: enzymes which are involved in inflammation through participation in pro- and anti-inflammatory mediators production and have an impact on many immunocompetent cells. The 30 members of the A_2_ superfamily are divided into 7 groups. Their role in asthma and COPD has been studied *in vitro* and *in vivo* (animal models, cell cultures, and patients). This paper contains complete and updated information about the involvement of particular enzymes in the etiology and course of asthma and COPD.

## 1. Introduction

Both asthma and COPD are airway diseases characterized by impaired airflow in the respiratory tract, chronic airway inflammation, as well as symptoms such as coughing, dyspnea, and wheezing. Intensive studies focused on the pathogenesis of these conditions implicate, among others, the group of phospholipases A_2_, which possess enzymatic and nonenzymatic properties. This paper presents general information about phospholipases and details the current knowledge about particular phospholipases A_2_ involved in asthma and COPD in human and animal models. The data regarding interactions between members of this superfamily is summarized, as well as the role of these enzymes in exacerbations of inflammatory diseases.

## 2. Phospholipases

**Figure 1:**
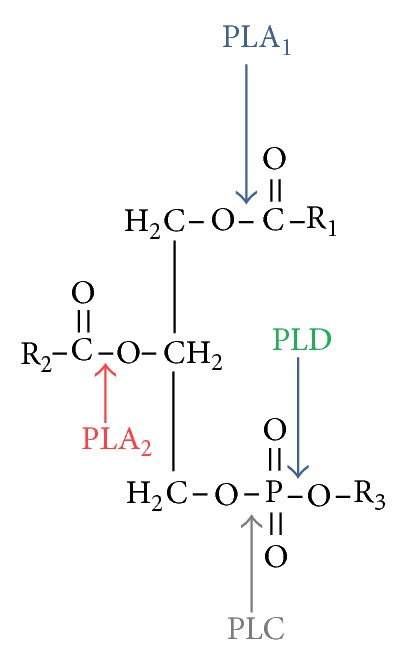
Phospholipases and their role in lipids metabolism.

Phospholipases are enzymes that hydrolyze phospholipids. The main substrates for these enzymes are glycerophospholipids which contain glycerol with a saturated fatty acid in the *sn-1* position and an unsaturated fatty acid in the *sn-2* position. The phospholipases responsible for hydrolysis of glycerophospholipids are divided into two groups: acylhydrolases and phosphodiesterases. The first group comprises phospholipase A_1_ (PLA_1_) and A_2_ (PLA_2_), which hydrolyze the ester bond at the *sn-1* and *sn-2* positions, respectively. The second group comprises phospholipase C (PLC) which cleaves the glycerol-phosphate bond, and phospholipase D (PLD), which liberates phosphatidic acid and alcohol (Figure 1). Phospholipase B shares both the properties of PLA_1_ and PLA_2_.

The structure, function, and catalytic mechanism of the enzyme determine its place within the phospholipase A_2_ superfamily, be it secretory PLA_2_ (sPLA_2_), cytosolic PLA_2_ (cPLA_2_), Ca^2+^-independent phospholipase A_2_ (iPLA_2_), PAF acetylhydrolases (PAF-AH), or lysosomal PLA_2_ (LPLA_2_). The latest classification, based on genetic structure, divides these enzymes into groups from I to XVI (in each one, the enzyme is represented by a capital letter) [1]. The characteristic features of each group are presented in Table 1.  Table 2 includes information about the mechanism of action and function of particular subgroups of PLA_2_s concerning physiology and pathophysiology.

## 3. Asthma and COPD

Currently about 300 million people worldwide suffer from asthma, and in 2025, this number is expected to grow by another 100 million. Annually, about 250 000 people die from asthma [2]. Asthma is defined according to the GINA (Global Initiative for Asthma) [3] as a chronic airway inflammatory disease in which many cells and cellular elements are involved. Chronic inflammation is a cause of bronchial hyperresponsiveness, leading to recurrent episodes of wheezing, dyspnea, chest tightness, and coughing, occurring particularly at night or dawn. This is usually accompanied by episodes of diffuse bronchial obstruction of varying severity, which often subside spontaneously or with treatment.

According to GOLD (The Global Initiative for Chronic Obstructive Lung Diseases) [6], COPD is characterized by a progressive and poorly reversible airflow limitation caused by both small airway diseases (airway inflammation and destruction) and parenchymal destruction (loss of alveolar attachment and decrease of elastic recall). Also, other extrapulmonary effects, such as weight loss, nutritional abnormalities, skeletal muscle dysfunction influence the severity of the disease. Apart from the genetic background (hereditary alpha-1 antitrypsin deficiency) [7] cigarette smoke is a crucial environmental factor in COPD development [8]; it is responsible for airway inflammation and further oxidant/antioxidant imbalance (oxidative stress) causing amplification of lung inflammation.

## 4. Analysis of Phospholipases A_**2**_ Involvement in Asthma and COPD

An analysis of studies concerning the profile of PLA_2_s expression in many experimental systems has revealed ambiguous results. Many different inductors used for cells stimulation cause expression of various types of enzymes in the same cells. Also, the presence of heterogeneous cells in experimental systems influences the expression of PLA_2_s [9].

Mast cells, Th_2_ lymphocytes, and eosinophils are the most important cellular components of asthma. It has been established that primary human lung mast cells constitutively express mRNA for the IB, IIA, IID, IIE, IIF, III, V, X, XIIA, and XIIB sPLA_2_ groups and stimulation with anti-IgE antibodies can induce their secretion [10]. Hence sPLA_2_ proteins are believed to belong to preformed mediators which are stored in mast cells granules. Cells stimulation by anti-IgE antibodies causes degranulation of mast cells, and sPLA_2_ appears in the early phase of allergic reaction. Muñoz et al. have shown that sPLA_2_V is not expressed in eosinophils in detectable amounts. However exogenous hPLA_2_V can activate eosinophils, inducing the liberation of arachidonic acid (AA) and LTC_4_ production [11]. Increased cPLA_2_ *α* phosphorylation and cPLA_2_ *α* activity was observed in eosinophils of asthmatics after allergen challenge [12].

Alveolar macrophages and neutrophils play a crucial role in the pathophysiology of COPD [13, 14]. Human macrophages express cPLA_2_IVA, iPLA_2_VIA, and several sPLA_2_s (IIA, IID, IIE, IIF, V, X, and XIIA, but not group IB and III enzymes). Higher expression of sPLA_2_IIA is observed after LPS treatment [15]. Neutrophils stimulated *in vitro* by the tripeptide formyl-Met-Leu-Phe (fMLP) demonstrate mRNA and protein expression of sPLA_2_V and sPLA_2_X, where the sPLA_2_V protein is found in azurophilic and specific granules, and sPLA_2_X is found only in azurophilic granules. GIB, GIIA, GIID, GIIE, GIIF, GIII, and GXII sPLA_2_s are undetectable. Cell activation by fMLP or zymosan results in the release of GV but not GX sPLA_2_ [16].

The BALF of patients with COPD demonstrates a three- to fivefold higher activity of PLA_2_s in comparison to a control BALF but the protein level shows no difference [17]. No differences in sPLA_2_IIs serum levels exist between healthy smokers and nonsmokers. However, significantly greater levels of this enzyme are found in the BALF of smokers compared with nonsmokers [18]. Among sPLA_2_s, sPLA_2_IID is also considered as a molecule involved in the course of COPD. A change of Gly80Ser in the sPLA_2_IID protein may be associated with body weight loss in patients suffering from COPD [19]. sPLA_2_IID can be also involved in control of inflammation by inhibition of CD4+, CD8+ T cells proliferation and induction of regulatory T cell differentiation [20]. Cigarette smoke extract (CSE) can induce the production of cytosolic phospholipase A_2_ in human pulmonary microvascular endothelial cells [21]. Moreover oxidative stress can increase the activity of cPLA_2_ by promoting its phosphorylation [22]. cPLA_2_ also participates in phosphodiesterase 4 signaling, whose inhibition attenuates neutrophilic inflammation in COPD [23]. The increased values of PLA_2_VII in patients with long-standing pulmonary hypertension (severe complication in COPD) are related to severe endothelial dysfunction [24].

sPLA_2_V plays a different role in the activation of eosinophils and neutrophils. Hence, its involvement in the pathogenesis of asthma and COPD can vary. Exogenous sPLA_2_V can activate the production of AA and leukotrienes in both cell types. However, LTB_4_ is preferentially produced in neutrophils, and LTC_4_ in eosinophils [11]. The sPLA_2_V-induced activation of neutrophils in contrast to eosinophils requires the presence and activation of cPLA_2_ [25]. The inhibition of cPLA_2_ may be more effective in diseases where neutrophils play a crucial role because they indirectly inhibit also the function of sPLA_2_.

## 5. Role of PLA_**2**_s in Asthma and COPD

The proposed mechanism of action of phospholipases A_2_ (PLA_2_s) in inflammatory diseases includes the liberation of arachidonic acid, generation of lysophospholipids, interaction between enzymes belonging to the A_2_ superfamily, surfactant degradation, release of cytokines, and the impact on immunological and inflammatory cells (dendritic cells, T-cells, and leukocytes) [26].

### 5.1. The Enzymatic Activity of PLA_2_s

The enzymatic properties of PLA_2_s refer to their phospholipase, lysophospholipase, transacylase, adiponutrin-like, triglyceride lipase, peroxiredoxin 6, and acyl-ceramide synthase activities. Phospholipases A_2_ play a pivotal role in eicosanoid production because they hydrolyze the ester bond at the *sn-2* position of the glycerophospholipid membrane, releasing arachidonic acid (AA) and lysophospholipids [27]. Arachidonic acid plays a dual role. It can act as a signaling molecule that regulates the activity of protein kinase C (PKC) and phospholipase C*γ*, influences Ca^2+^ concentration, and acts as an endogenous ligand for PPAR*γ* receptors [28, 29]. AA is also a precursor of lipid inflammatory mediators (eicosanoids). In cyclooxygenase (COX) pathways, it is transformed to prostaglandins and thromboxane while in lipoxygenase (ALOX) pathways, it is converted to leukotrienes. These molecules are responsible for bronchial constriction, increased vessel permeability, and inflammatory cell recruitment [30]. AA is also a substrate for resolvins and lipoxins (LXs) which have anti-inflammatory properties. Lipoxins can block granulocyte chemotaxis, migration, degranulation, oxidative burst, cytokine-mediated signaling in eosinophils, and secretion of cytokines from bronchial epithelial cells [31]. Several independent studies have reported that significantly lower levels of LXs are observed in severe asthmatics compared to patients with nonsevere asthma [32, 33]. Resolvins demonstrate endogenous anti-inflammatory, proresolving, antifibrotic, antiangiogenic, anti-infective, and antihyperalgesic activity [31].

Among cytosolic phospholipases A_2_, it has been well documented that cPLA_2_IVA (cPLA_2_ *α*) plays an important role in eicosanoid production. In patients with inherited cPLA_2_ deficiency (loss-of-function mutations in both cPLA_2_ alleles), a widespread decrease in eicosanoid concentrations has been observed [34]. S111P, R485H, and K651R mutations in *PLA2G4A* gene are thought to play a crucial role in this condition. The functional consequences of localized mutations concerning cPLA_2_ catalytic activity, Ca^2+^ recruitment, and affinity for the phospholipid membrane have been confirmed *in vitro* and in cell culture [35]. In patients with severe asthma, the microsatellite fragments (T)_n_ and (CA)_n_ in the promoter region of cPLA_2_ *α* gene (*PLA2G4A*) are shorter in comparison to healthy subjects [36]. In addition, asthmatic patients with shorter microsatellite sequences demonstrate greater expression of cPLA_2_ *α* mRNA, cPLA_2_ *α* protein, PGE_2_ and 15-HETE, but not LTC_4_ [37]. cPLA_2_ participates in intracellular signaling, leading to allergen-induced production of inflammatory cytokines in the PBMC of asthmatics [38]. Hallstrand et al. [39] identified increased expression of three cPLA_2_s, including cPLA_2_ *α*, cPLA_2_ *β*, and cPLA_2_ *γ* in induced sputum cells from subjects with asthma and exercise-induced bronchoconstriction. Both cPLA_2_ *β* and cPLA_2_ *γ* enzymes also participate in eicosanoids biosynthesis [40, 41]. Increased cPLA_2_ expression and subsequent PGE_2_ production are present in the asthma phenotype. The therapeutic decision to inhibit cPLA_2_ in asthmatics may be unclear when considering the role of PGE_2_ in airway inflammation. There is some evidence that PGE_2_ can act as bronchodilator, as well as an inhibitor of both allergen-induced bronchoconstriction and inflammatory mediators production [42]. It should be noticed that PGE_2_ acts through four different types of receptors (EP_1_, EP_2_, EP3, and EP_4_). Changes in expression and combination of receptor subtypes actions may affect the action of PGE_2_ giving it proinflammatory or bronchoprotective outcomes [43–45]. The pleiotropic properties of PGE_2_ make it difficult to establish the direct impact of PGE_2_ deficiency which appears as a consequence of cPLA_2_ inhibition [46]. Moreover, although cPLA_2_ is a major enzyme, it is not the only one providing substrates for eicosanoids synthesis; hence it cannot be excluded that other existing pathways can also perform this function.

sPLA_2_s and arachidonic acid accumulate in the BALF of asthmatics after allergen challenge [47, 48]. Despite being specific to the *sn-2* bond, sPLA_2_s play more of a supporting role in AA liberation. Only sPLA_2_V and sPLA_2_X can efficiently interact and hydrolyze phospholipids from the outer surface of the cell membrane [9]. In acute and chronic animal asthma models, a deficit of sPLA_2_X diminishes the features of asthma (eosinophilia, airway hyperresponsiveness to methacholine, airway remodeling, eicosanoids, and Th2 cytokine production) [49].

Hallstrand et al. [50] showed that the expression of sPLA_2_X predominates in the airway epithelium, and both sPLA_2_X and sPLA_2_IIA are the main phospholipases produced by BALF cells. The activity of the sPLA_2_V protein was found to be greatly lowered and undetectable. They have suggested that sPLA_2_X is most important among secretory phospholipases. Only sPLA_2_X, not sPLA_2_IIA, is correlated with asthma features such as lung function, recruitment of neutrophils in asthmatics [50]. sPLA_2_X is responsible for production of cysteinyl leukotrienes (cysLTs) which are proinflammatory in asthma and can be responsible for observable features of asthma. Moreover, the level of prostaglandin E_2_ (PGE_2_) is also connected with sPLA_2_X, which can be explained by the fact that sPLA_2_X increases activity of cPLA_2_IV which in turn leads to production of PGE_2_. These results are consistent with earlier studies by the same authors in which gene expression of sPLA_2_X and sPLA_2_ XII was demonstrated to be elevated in induced sputum cells of patients with asthma. The level of sPLA_2_X in induced sputum cells supernatant increased after exercise challenge among asthmatics with exercise-induced bronchoconstriction (EIB) [39]. Lai et al. [51] have confirmed the involvement of sPLA_2_X. They demonstrated that recombinant sPLA_2_X caused AA release and rapid onset of cysLT synthesis in human eosinophils.

Limited information suggests a possible anti-inflammatory role of sPLA_2_X. However in asthma, sPLA_2_X facilitates the polarization toward proasthmatic M2-macrophage phenotype [52]. It is possible that in a proinflammatory environment, that the sPLA_2_X propeptide is more rapidly converted to an active form that might influence the Th1/Th2 balance [53]. All these factors may suppress its anti-inflammatory action.

Other sPLA_2_s (IIA, IID, IIE) contain a heparin-binding domain which allows these enzymes to be taken into the cells and further directed to compartments enriched in AA and enzymes responsible for eicosanoid production [54].

In spite of the fact that several studies have confirmed the participation of iPLA_2_ *β* [55] and iPLA_2_ *γ* [56] in AA release and eicosanoid production, there is no data indicating that these enzymes play a direct role in asthma. By the induction of Ca^2+^ influx they can influence the translocation and activity of Ca^2+^-dependent PLA_2_s isoforms.

Group VII and VIII PAF-AH hydrolyze the short *sn-2* residue of PAF (platelet activating factor). As they lack activity against membrane phospholipids with long-chain *sn-2* residues, they are unable to release arachidonic acid from membrane phospholipids [57]. They exhibit pro- and anti-inflammatory properties. On the one hand, they inactivate PAF—the proinflammatory mediator—by hydrolyzing it to inactive acetate and lysolipid but on the other hand, they assist in the generation of lysophospholipids and fatty acid hydroperoxides [4]. Stafforini et al. [58] have established that asthmatics have a decreased level of PAF-AH, and that asthma incidence and severity correlate to PAF-AH deficiency in the Japanese population. Also some *PAF-AH* gene polymorphisms (Ile198Thr and Ala379Val variants) are known to be a risk factors for developing atopy and asthma [59]. Despite positive effects in animal models [60], administration of human recombinant PAF-AH (rPAF-AH) does not reduce both early and late phase of asthmatic response in mild asthmatics challenged with allergens [61].

The enzymatic activity of PLA_2_s embraces also lysophospholipid generation. Lysophospholipids are biologically active molecules acting through specific receptors. They are a precursor of platelet activating factor (PAF) and lysophosphatidic acid (LPA). LPA is involved in cell adhesion, motility, and survival. In animal models, lysophospholipid receptors are required for proper development and function of the cardiovascular, immune, respiratory, and reproductive systems [62]. Lysophosphocholine and polyunsaturated fatty acids, including AA, can activate cPLA_2_ and 5-lipoxygenase by increasing Ca^2+^ and inducing cPLA_2_ phosphorylation, which then leads to LTB_4_ biosynthesis [25]. Lysophospholipid has nonspecific cytotoxic effect that depends on its concentration (critical micelle concentration). At concentration below their unspecific cytotoxic effect lysophospholipids can induce apoptosis by interrupting the synthesis of phosphatidylcholine [63].

Phospholipases A_2_ activity is also connected with disturbed lipid homeostasis in the lung. Asthma and other inflammatory lung diseases are characterized by impaired surfactant function [64]. Secretory phospholipases degrade phosphatidylcholine (PC), the main component of the surfactant responsible for maintenance of small airway patency. The generation of lysophospholipids and free fatty acids by sPLA_2_-mediated PC hydrolysis has been implicated in small airway closure in asthm. sPLA_2_ action is enhanced by eosinophilic lysophospholipases that use lysophospholipids as a substrate [65–68]. The presence of iPLA_2_ proteins in alveolar macrophages suggests that they might play a role in surfactant degradation [69].

It should be mentioned that some PLA_2_s are involved in antibacterial defense thanks to their ability to hydrolyze the lipids of the bacterial membrane. sPLA_2_s IIA, V, X, and IB demonstrate bactericidal activity against gram-positive pathogens but the most effective is sPLA_2_IIA. Group XII can directly kill *E.coli*, unlike the other sPLA_2_s that require cofactors [70]. This property of phospholipases can be important in bacterial exacerbations of asthma and COPD.

### 5.2. Nonenzymatic Activity of PLA_2_s

The secretory forms of many PLA_2_s exert a range of actions in airway inflammation. Apart from their enzymatic activity, they can act as extracellular mediators involved in chemotaxis, cytokine production, and induction of cellular signaling pathways.

Mammalian N-type receptors have been identified for sPLA_2_IB and IIA, X and M-type receptors for sPLA_2_IB, IIA, IIE, IIF, V, and X [71]. N-type like receptors are present in lungs whereas M-type receptors have been identified in lung and myeloid cells [72]. The binding of sPLA_2_s to their M-type receptor deactivates their enzymatic properties [73].

sPLA_2_s are stored in intrinsic mast cell granulates and are released after cell activation by IgE and non-IgE stimuli [9]. After exocytosis, they can act in both autocrine and paracrine manners. By interacting with heparan sulphate proteoglycans and M-type receptors, they can induce PGD_2_ and LTC_4_ production and stimulate the subsequent degranulation of mast cells [74]. Granata et al. [17] delivered an evidence that sPLA_2_s can act as proinflammatory connections between mast cells and macrophages in the airway. They suggest that the activation of macrophages by sPLA_2_s leads to production of proinflammatory cytokines which sustain the inflammatory and immune response, chemokines responsible for recruitment of monocytes and neutrophils, as well as destructive lysosomal enzymes, NO, PGE_2_, and metalloproteinases connected with airway remodeling [17]. The sPLA_2_s induce *β*-glucuronidase release and production of IL-6 from human lung macrophages [75]. They influence the migration and adhesion of neutrophils as well as the release of elastase [76, 77]. In eosinophils, sPLA_2_ IA and IIA stimulate *β*-glucuronidase release and cytokine production (IL-6, IL-8) by AA and lysophospholipid generation, by interaction with membrane peptidoglycans via their heparin-binding site, and through binding with specific M-type or N-type receptors [78]. The functions of sPLA_2_s receptors require further studies because there are still some missing or unequivocal information [52].

### 5.3. Crosstalk between PLA_2_s

The phospholipases can cooperate in mechanism leading to eicosanoid production. sPLA_2_ and cPLA_2_ interaction is quite well documented [79, 80]. The effect of group IIa and V PLA_2_s on H_2_O_2_-induced AA release is dependent upon the presence of cPLA_2_ and the activation of PKC and ERK1/2 in murine mesangial cells. Offer et al. [81] have described negative feedback between sPLA_2_ and cPLA_2_ in eicosanoid production. sPLA_2_ activation induces production of bronchoconstrictor cysteinyl leukotrienes and suppresses cPLA_2_ expression and the subsequent production of bronchodilator PGE_2_. Recently it has been established that in human eosinophils, sPLA_2_ initiates Ser(505) phosphorylation of cPLA_2_ *α* and stimulates leukotriene synthesis through involvement of p38 and JNK MAPK, cPLA_2_ *α*, and 5-lipoxygenase activation, which may be an important process also in airways of asthmatics [51]. Also in bone-marrow-derived mast cells, sPLA_2_ mediates the selective release of AA by binding M-type receptors and then inducing MAPK signaling pathways that lead to cPLA_2_ activation [82].

### 5.4. PLA_2_s in the Exacerbation of Disease

Another aspect of phospholipases and the asthma/COPD relationship is the participation of these enzymes in the pathogenetic mechanisms of disease exacerbation caused by bacterial factors. This role relates to increased expression of selective PLA_2_s, modulation of their activity and involvement in cellular signaling. Elevated cPLA_2_ *α* expression was found in primary human lung macrophages after LPS treatment [15, 83]. LPS stimulates expression of cPLA_2_ and COX-2 in macrophages, leading to increased production of AA and PGE_2_ [83]. LPS treatment was also followed by rapid changes in cPLA_2_ phosphorylation [84, 85]. This is one of the mechanisms of regulating enzyme activity [86]. The LPS-phosphorylated form of cPLA_2_ is present in induction of iNOS and TNF-*α* expression [87, 88] and metalloproteinase production [89]. Selective sPLA_2_ contributes to LPS-intracellular signaling in liver macrophages [84, 90, 91].

In mice with LPS-induced lung inflammation, the expression of sPLA_2_X remains the same before and after treatment. In this study, increased expression of sPLA_2_IID and sPLA_2_V has been observed, as well as decreased sPLA_2_IIE and sPLA2IIF levels in the lungs. In rats, sPLA_2_IIA was seen to have the highest expression after LPS administration [92]. In msPLA_2_X^−/−^ mice with knock-in of human sPLA_2_X (hsPLA_2_X), allergen-induced inflammatory cell recruitment into airways (eosinophils) was restored, as well as hyperresponsiveness to methacholine. The application of specific hsPLA_2_X inhibitor (RO 061606) significantly attenuates airway inflammation symptoms, mucous secretion, and hyperresponsiveness [93]. In sPLA_2_V^−/−^ knock-out mice, sPLA_2_V has been proven to play a role in the development of lung injury and neutrophilic inflammation after bacterial stimulus (LPS) [94]. In addition, sPLA_2_V was seen to be connected with regulation of cell migration and generation of airway hyperresponsiveness after ovalbumin challenge [95]. In a murine allergen-challenged asthma model, administration of rPAF-AH is effective in blocking late-phase pulmonary inflammation [60].

## 6. The Clinical Significance of Studying the Participation of PLA_**2**_s in Airway Inflammatory Diseases

Taking into consideration the severe asthma phenotype, the difficulties related to obtain asthma control utilizing currently available treatments and the progressive character of inflammation in patients with COPD that increases the morbidity, it seems reasonable to study the differences in pathogenesis of the diseases conditions, especially in relation to possible new therapies and drugs. The PLA_2_s are an interesting object of study for several reasons. The superfamily of these enzymes contains approximately 30 members that have similar and isoform-specific properties. It has been confirmed that they are strictly connected with inflammation. The inhibitors of particular PLA_2_s show the positive effect in treatment of inflammatory diseases [96] and they inhibit allergic reaction *in vitro* [38]. The cPLA_2_ *α* that evolved together with receptors for eicosanoids, present only in vertebrate, seems to play crucial role in course of inflammation. Its inhibitors such as efipladib [97] and ecopladib [98] successfully inhibit inflammation in rheumatoid arthritis and osteoporosis. The inhaled form of cPLA_2_ *α* inhibitor, the PLA-950, is considered as potential new treatment in asthmatic patients as well as other PLA_2_s can influence the function of cPLA_2_ *α* or have similar effects. Recent studies report positive results of a preclinical evaluation of a cPLA_2_ *α* inhibitor [99]. The studies and analysis of protein involved in regulation of particular sPLA_2_ involved in inflammatory diseases could result in finding new target for drugs.

Since 1980, it has been known that glucocorticoids (GCs) can inhibit the activity of PLA_2_ [100]. The underlying mechanism concerns induction of mRNA and protein expression of lipocortin 1 (annexin 1) and the PLA_2_ inhibitory protein [101–104]. The structure, function, and mechanism behind the anti-inflammatory action of annexin 1 have been well described elsewhere [105]. Glucocorticoids can also suppress the production of sPLA_2_IIA by blocking mRNA synthesis and posttranslational expression in rats [106]. It is questionable whether therapeutic doses of glucocorticoids have sufficient power to satisfactorily inhibit the activity of PLA_2_. Juergens et al. [107] demonstrated that topical GCs at therapeutically relevant concentration (10^−8^ M) inhibit the spontaneous activity of cPLA_2_ in the range of 8.6–17.3% depending on the type of GC. They suggest also that this effect may appear as a consequence of a decreased ability to binding the receptors by GCs present in airway in subtherapeutical doses. Although it has been established that treatment with GCs can indirectly inhibit cPLA_2_ and AA-derivates production resistance to GCs in patients with asthma and COPD could also be problematic. Moreover the GCs have systemic effects and long-term application can cause the side effects. The approach to attack the inflammation process more precisely and downstream (inhibition the eicosanoids production) seems to be rationale.

Another aspect regarding annexin 1 and PLA_2_s is their cell-specific manner of interactions [105]. Kwon et al. [108] demonstrated that cleavage of annexin 1 causes phosphorylation of cPLA_2_ during mast-cell activation. Hence it is not clear whether GCs-induced expression of annexin always leads to inhibition of cPLA_2_ activity. Posttranslational changes can dramatically influence the primary protein function. As previous studies indicate that GCs can stimulate expression of cPLA_2_ in amnion fibroblast it cannot be excluded that in some specific circumstances GCs may directly induce cPLA_2_ [109, 110].

## 7. Conclusions

Previous studies confirm the involvement of phospholipases A_2_ in asthma and COPD although there are some gaps relating to the roles of specific enzymes. The participation of PLA_2_ in asthma pathogenesis has been better investigated. The diagnostic problems concerning the overlap syndrome that shares the features of asthma and COPD demand further studies on the pathogenesis of these diseases. The phospholipases A_2_ through their involvement in the course of inflammation seem to be important aspects of this investigation. As they demonstrate pro- and anti-inflammatory properties, a detailed analysis of their role should act as a focus for further studies intended to bring new insights into the pathogenesis of the diseases and identify targets for new drugs.

Data from studies focused on role of PLA_2_s in inflammatory diseases facilitate the understanding of molecular aspects of inflammation. It can be observed that cPLA_2_ plays a main role in eicosanoid production and other PLA_2_s may influence their activity thanks to enzymatic properties or act as regulators of inflammation through their nonenzymatic activity. The pleiotropic properties of single phospholipase and their differential expression in many cells confirm that this is well-organized network of interaction, and further studies focused on this aspect may provide more useful knowledge. A comparison of how this network works in different inflammatory diseases, as well as in healthy subjects may indicate a key molecule, whose activity or presence will be a diagnostic parameter or whose activation or inhibition will have therapeutic value.

Asthma and COPD are heterogeneous diseases and current treatment gives only the possibility to obtain the phenotype of well-controlled diseases. Analysis of data regarding the involvement of PLA_2_s in course of diseases arises the concept to use combined therapy rather than the treatment based on inhibition of one of them. The results from preclinical studies of cPLA_2_ inhibitors are promising but clinical trials will give concrete knowledge about the effectiveness and possible side effects.

## Figures and Tables

**Table 1:** Characteristics of structure and localization of human phospholipase A_2_ enzymes. Adapted and modified from [1, 4]. The Roman numeral indicates the group, and the capital letter after the number indicates the subgroup.

Name	Members (human)	Molecular mass (kDa)	Relationship with Ca^2+^	Catalytic site	Localization
Secretory phospholipase A_2 _(sPLA_2_)	IB (sPLA_2_IB)	13–15			Secreted
	IIA (sPLA_2_IIA)	13–15			Secreted; membrane; secretory granules
	IID (sPLA_2_IID)	14-15			Secreted
	IIE (sPLA_2_IIE)	14-15			Secreted
	IIF (sPLA_2_IIF)	16-17			Secreted
	III (sPLA_2_III)	55	Dependent	Histidine/Aspartic acid	Secreted
	V (sPLA_2_V)	14			Secreted; Golgi apparatus; nuclear envelope; plasma membrane
	X (sPLA_2_X)	14			Secreted
	XIIA (sPLA_2_XIIA)	19			Secreted; cytoplasm
	XIIB (sPLA_2_XIIB, XIII)	20			Secreted

	IVA (cPLA_2_ *α*)	85	Dependent		Nucleus; cytoplasmic vesicles
	IVB (cPLA_2_ *β*)-three splice variants	114			Cytosol
Cytosolic phospholipase A_2 _(cPLA_2_)	IVC (cPLA_2_ *γ*)	61	Independent		ER; Mitochondrium
	IVD (cPLA_2_ *δ*)	92-93		Serine/Aspartic acid/Arginine	Cytosol; Cytoplasmic vesicle membrane; peripheral membrane protein; cytoplasmic side
	IVE (cPLA_2_ *ε*)	96	Dependent		Cytosol; lysosome membrane; peripheral membrane protein
	IVF (cPLA_2_ *ζ*)	95			Cytosol; lysosome membrane; peripheral membrane protein; cytoplasmic side

Ca^2+^-independent phospholipase A_2 _(iPLA_2_)	VIA-(iPLA_2_ *β*)-five splice variants	84–90			Cytosol
	VIB (iPLA_2_ *γ*)-four splice variants	88–91			ER; peroxisomal and mitochondrial membrane
	VIC (iPLA_2_ *δ*, NTE)	146	Independent	Serine	ER; single-pass type I membrane protein; cytoplasmic side
	VID (iPLA_2_ *ε*, adiponutrin)	53			Membrane; single-pass type II membrane protein
	VIE (iPLA_2_ *ζ*)	57			Lipid droplet membrane; single-pass type II membrane protein; cell membrane
	VIF (iPLA_2_ *η*)	28			Cytoplasm

Acidic Ca^2+^-independent phospholipase A_2_	aiPLA_2_	26	Independent	Serine	Cytoplasm; Lysosome

Lysosomal phospholipase A_2 _	XV (LPLA_2_, LLPL, ACS)	45	Independent	Serine/Histidine/Aspartic acid	Secreted; Lysosome

PAF acetylhydrolase (PAF-AH) or Lipoprotein-associated phospholipase A_2_	VIIA (Lp-PLA_2, _Plasma PAF-AH)	45	Independent	Serine/Histidine/Aspartic acid	Secreted
	VIIB (PAF-AH II)	40			Cytoplasm
	VIIIA (PAF-AH Ib) *α*1 subunit	26			Cytoplasm
	VIIIB (PAF-AH Ib) *α*2 subunit	26			Cytoplasm

Adipose-specific phospholipase A_2_	XVI (H-Rev107)	18	Independent	Cystein/Histidine/Histidine	Cytoplasm, perinuclear region, Single-pass membrane protein

ER: endoplasmic reticulum; NTE: neuropathy target esterase.

**Table 2:** Mechanism of action and function of human phospholipase A_2_ enzymes. Adapted and modified from [1, 4, 5].

Name	Mechanism of action	Function		Sources
		Physiology	Pathophysiology
Secretory phospholipases A_2 _(sPLA_2_s)	(i) Enzymatic (liberation of AA and lysophospholipids) (ii) Autocrine and paracrine action by binding to N-type and M-type receptors or by binding to integrins	(i) Lipid remodeling for membrane homeostasis (ii) Exocytosis (iii) Phagocytosis (iv) Anticoagulant activity (v) Antibacterial activity (Gram-positive and Gram-negative bacteria) (vi) Antifungal and antiadenoviral activity (vii) Parturition (viii) Spinal processing of nociception	(i) Inflammatory diseases (rheumatoid arthritis, adult respiratory distress syndrome, inflammatory bowel disease, and pancreatitis) (ii) Sepsis (iii) Atherosclerosis (foam cell formation) (iv) Cancer (v) Surfactant hydrolysis	Neutrophils, eosinophils, basophils, T-cells, monocytes, macrophages, platelets, mast cells, airway epithelial cells, alveolar type II epithelial cells,

Cytosolic phospholipases A_2 _(cPLA_2_s)	(i) enzymatic: lysophospholipase and transacylase activity	(i) AA releasing (ii) Cellular signaling (iii) Parturition (iv) Nociception	(i) Inflammation (ii) Intestinal ulceration (iii) Psoriasis (iv) Acute lung injury (v) Polyposis (vi) Brain injury (vii) Anaphylaxis	Every tissue

Ca^2+^-independent phospholipases A_2 _(iPLA_2_s)	VIA, VIB, VIC, VID, VIEVIF-phospholipase A_2_ activity VIC-lysophospholipase activity VID-adiponutrin-like activity VIE-triglyceride lipase activity VIF-transacylase activity	(i) Remodeling of phospholipids (ii) AA releasing (iii) Protein expression (iv) Acetylcholine-mediated endothelium-dependent relaxation of the vasculature (v) Apoptosis (vi) Insulin secretion (vii) Bone formation (viii) Sperm development (ix) Cell proliferation (x) Activation of Ca^2+^ influx (xi) Axon regeneration in nerve injury (VIA)	(i) Wallerian degeneration (VIA) (ii) regulation of monocyte migration (VIB) (iii) Oxidant-induced cell injury (VIC) (iv) Ischemia-induced ventricular tachyarrhythmias	(i) Alveolar cells (ii) Macrophages (iii) Normal and cancer lung tissue (iv) Neurons
	aiPLA_2_-phospholipase A_2_ and peroxiredoxin 6 activity	(i) Degradation and recycling of surfactant phospholipids (remodeling of phosphatidylcholine to dipalmitoyl-phosphatidylcholine (DPPC) (ii) Antioxidative activity	(i) lung cancer, mesothelioma, sarcoidosis	(i) Alveolar macrophages (ii) Type II epithelial cells (iii) Clara cells

Lysosomal phospholipase A_2 _	(i) Acyl-ceramide synthase (ii) Transacylase activity (iii) Lysophospholipase activity	(i) may be the crucial enzyme of pulmonary surfactant phospholipid degradation by alveolar macrophages	(i) Phospholipidosis (ii) Complement activation (iii) Induced lung injury	(i) Alveolar macrophages (ii) Peripheral blood monocytes

PAF acetylhydrolases (PAF-AH) or Lipoprotein-associated phospholipases A_2_	(i) Phospholipase A_2_ activity	(i) Anti-inflammatory properties by hydrolyzing platelet activating factor (PAF) (ii) Protection against oxidative stress (iii) Brain development	(i) Generation of lysophospholipids and fatty acid hydroperoxides (ROS) (ii) Acute respiratory distress syndrome (iii) Marker of coronary heart disease (iv) Miller-Diker lissencephaly	(i) Alveolar macrophages (ii) Epithelial type II cells

Adipose-specific phospholipase A_2_	(i) Phospholipase A_1_ and A_2_ activity	(i) catalyzes the release of fatty acids from phospholipids in adipose tissue	(i) Obesity (ii) Metabolic syndrome	Adipose tissue
